# Oral health considerations in patients receiving antidepressant therapy: Clinical implications and management strategies

**DOI:** 10.6026/973206300221046

**Published:** 2026-02-28

**Authors:** Pihu Jamwal, PK Monika, Aditi Pustake, Nisha Dholakiya, Roshani Shrestha, Ketki Joshi

**Affiliations:** 1 Department of Dentistry, Institute of Dental Sciences, Jammu & Kashmir, India; 2 Department of Prosthodontics, Lenora Institute of Dental Sciences, Rajahmundry, Andhra Pradesh, India; 3 Department of Dentistry, Sharad Pawar Dental College and Hospital, Sawangi (Meghe), Wardha, Maharashtra, India; 4 Department of Dentistry, College of Dental Science and Research Center, Gujarat, India; 5 Department of Dentistry, Nepal Medical College and Teaching Hospital, Gokarneshwar, Kathmandu, Nepal; 6 Department of Dentistry, Tatyasaheb Kore Dental College and Research Center, Maharashtra, India

**Keywords:** Antidepressants, xerostomia, dental caries, periodontal disease, oral health management

## Abstract

Patients receiving antidepressant therapy frequently present with oral health complications that are under recognized and inadequately addressed in routine dental practice. Antidepressant medications-particularly selective serotonin reuptake inhibitors (SSRIs) and tricyclic antidepressants (TCAs)-are widely prescribed and have significant implications for oral health. Their adverse effects include xerostomia, increased caries risk, periodontal disease and bruxism and delayed wound healing, while depression itself may contribute to poor oral hygiene practices and irregular dental attendance. Additionally, pharmacologic interactions with vasoconstrictors, analgesics, antibiotics and antifungals can complicate dental management, particularly in medically compromised and geriatric patients. This review integrates pharmacologic, behavioral and clinical considerations related to antidepressant use and translates existing evidence into practical strategies for comprehensive dental management, thereby providing a clinically relevant framework for dental practitioners.

## Background:

Antidepressant medications are among the most commonly prescribed psychotropic agents worldwide, with a growing proportion of dental patients receiving long-term therapy [1]. Recent evidence indicates that antidepressant use- particularly selective serotonin reuptake inhibitors (SSRIs) and tricyclic antidepressants (TCAs)-is associated with oral health complications such as xerostomia, increased caries risk and alterations in periodontal status, which may negatively influence dental treatment outcomes and patient quality of life [2, 3]. In addition, depression itself has been linked to poor oral hygiene practices and irregular dental attendance, further exacerbating oral disease risk [4]. Despite these recognized associations, guidance on the comprehensive dental management of patients receiving antidepressant therapy remains fragmented. Therefore, it is of interest to describe the oral health implications of antidepressant therapy and to outline evidence-based clinical management strategies relevant to dental practice.

## Antidepressants in dental patients:

## Scope and clinical significance:

The rising use of antidepressant medications has important implications for dental practice. According to a study in the Journal of the American Dental Association analyzing 1,800 patient records, 21% of patients were undergoing antidepressant therapy, most commonly with SSRIs, followed by TCAs, atypical antidepressants and monoamine oxidase inhibitors (MAOIs). More than half of these patients were prescribed medications associated with xerostomia and two-thirds (67%) were prescribed drugs linked to orthostatic hypotension [5]. The overlapping effects and interactions of these medications increase the risk of complications such as dry mouth, dizziness and vasoconstrictor sensitivity, necessitating tailored clinical approaches [5]. Emerging evidence also suggests potential therapeutic uses of antidepressants in dentistry. A systematic review of 15 randomized controlled trials reported effectiveness in treating acute and chronic pain, bruxism and burning mouth syndrome, with amitriptyline being the most frequently studied drug [6]. While methodological variability limits definitive conclusions, these findings provide a rationale for considering antidepressants in select areas of dental care [6]. Adverse oral effects remain well documented. A systematic review of 11 studies linked antidepressant use, particularly SSRIs and TCAs, to xerostomia, caries and periodontal disease [1]. Reduced salivary flow clearly contributes to higher caries risk, although no significant effects have been observed on saliva pH, chemical composition, or intraoperative hemostasis. Additional issues such as taste dysfunction and oral bleeding have also been reported [1]. The risk of oral bleeding with SSRIs appears minimal. A retrospective cohort study of 92 SSRI users undergoing 145 invasive dental procedures, including extractions, implant surgeries, periodontal interventions and biopsies, reported only one return visit and one phone consultation for postoperative bleeding, indicating that bleeding complications are rare and manageable. Among these complications, salivary gland hypofunction is particularly significant, representing one of the most consistent and clinically relevant side effects of antidepressant therapy [7]. Given its frequency and impact, salivary dysfunction is considered one of the most important oral complications of antidepressant use and is discussed next in detail.

## Salivary dysfunction and caries risk associated with antidepressant use:

Antidepressants, including selective serotonin reuptake inhibitors (SSRIs), tricyclic antidepressants (TCAs) and serotonin- norepinephrine reuptake inhibitors (SNRIs), are strongly associated with salivary gland hypofunction [8]. Reduced secretion produces xerostomia, impairs natural cleansing and buffering, diminishes antimicrobial protection and compromises tooth remineralization, thereby increasing caries risk [8]. TCAs such as amitriptyline exert pronounced anticholinergic effects, causing a marked decrease in salivary flow and impaired plaque clearance, whereas SSRIs, including fluoxetine and sertraline, exert milder anticholinergic activity but may suppress secretion via central autonomic pathways [9]. Fluoxetine has been shown to reduce unstimulated salivation in a dose-dependent manner, placing elderly patients at particular risk due to polypharmacy [9]. Persistent hyposalivation fosters a cariogenic environment by favoring Streptococcus mutans and Lactobacillus proliferation, leading to enamel demineralization and lesion progression [10]. Qualitative alterations in saliva, such as reductions in IgA, lysozyme and lactoferrin, further compromise host defenses. Clinical studies report higher DMFT scores in long-term SSRI users, reinforcing the link between antidepressant-induced xerostomia and caries [10]. Depression itself compounds this risk, as affected individuals may neglect oral hygiene, increase carbohydrate intake, or use tobacco and alcohol, further predisposing to caries and periodontal disease [11]. Preventive strategies include patient education, saliva substitutes or stimulants, topical fluorides and reinforcement of oral hygiene, along with regular dental monitoring [11]. In severe cases, collaboration with prescribing physicians to adjust therapy toward less xerogenic agents may be warranted [12]. Beyond caries, reduced salivary protection and drug effects also compromise periodontal tissues and wound healing [12], which are examined in the following section.

## Effect of antidepressants on periodontal health and wound healing:

Periodontal disease encompasses inflammatory conditions affecting the supporting structures of the teeth, including the gingiva, periodontal ligament, cementum and alveolar bone. It often begins as gingivitis; a reversible inflammation caused by plaque accumulation and may progress to periodontitis, characterized by tissue attachment loss, alveolar bone resorption and eventual tooth loss [13]. Anaerobic bacteria such as Porphyromonas gingivalis and Treponema denticola trigger immune responses that, while combating infection, can also damage surrounding tissues and accelerate disease progression [13]. Depression itself is an independent risk factor for periodontal disease due to poor oral hygiene, increased smoking, immune alterations and xerostomia, which promote pathogenic bacterial growth and tissue breakdown [14]. Antidepressants, including MAOIs, SSRIs, SNRIs and TCAs, may further impact periodontal health [15, 16]. Clinical studies indicate that venlafaxine is associated with worsened periodontal parameters, including increased gingival index, probing depth, attachment loss and alveolar bone inflammation [15]. SSRIs such as fluoxetine may induce movement-related side effects, including bruxism, which increases mechanical stress on periodontal tissues [14]. Wound healing is a complex, multi-step process involving fibroblast proliferation, collagen deposition, angiogenesis and platelet aggregation. Serotonin contributes to collagen synthesis, fibroblast growth and angiogenesis [17]. SSRIs reduce serotonin uptake and impair platelet aggregation by altering intracellular calcium mobilization, which may compromise hemostasis and tissue repair [17]. Preclinical and clinical studies suggest that SSRIs, including sertraline, may inhibit craniofacial bone healing and alter bone remodeling dynamics, highlighting the importance of monitoring periodontal and surgical outcomes in patients receiving these medications [18, 19].

## Oral health neglect in patients with depression (behavioral aspects):

Depression can make you feel sad all the time, lose interest in things, have low energy and have trouble concentrating. These symptoms can make it harder for people to keep their mouths clean, go to the dentist on time and follow through with the treatments that are suggested. Studies demonstrate that depressed individuals often skip tooth brushing and flossing, consume carbohydrate-rich foods and use tobacco or alcohol, all contributing to increased risk of caries and periodontal disease [20]. Systematic reviews report higher rates of untreated decay, missing teeth and edentulism in this population, as well as worse DMFT indices and periodontal parameters [21]. Dental care-seeking behaviors are also affected, with patients often delaying visits until urgent treatment is necessary, which can further exacerbate oral disease. This neglect negatively affects self-image and social functioning, perpetuating a cycle of depression and oral disease [22]. Effective strategies to address these challenges include screening for depressive symptoms using validated tools such as the PHQ-9 is a short, nine-question checklist that helps spot signs of depression. It asks about things like mood, sleep, energy, appetite and how much interest you've had in your usual activities over the past couple of weeks. Your answers add up to a score that gives a quick sense of whether you might be dealing with mild, moderate, or more serious depression. Structures supportive appointments that encourage adherence, reinforcing oral hygiene, preventive care and collaborating with mental health professionals [23]. By addressing both behavioral and biological factors, dental care providers can ensure comprehensive management and improve long-term oral health outcomes in patients with depression [16]. In addition to these behavioral influences, dentists must also be mindful of pharmacologic complexities, as antidepressants frequently interact with common dental agents. These interactions are reviewed in the next section [16].

## Pharmacologic considerations and drug interactions relevant to dentistry:

Antidepressants are broadly divided into five classes: tricyclic antidepressants (TCAs), selective serotonin reuptake inhibitors (SSRIs), monoamine oxidase inhibitors (MAOIs), serotonin-norepinephrine reuptake inhibitors (SNRIs) and atypical agents, each associated with distinct oral and systemic effects, including xerostomia, dysgeusia, impaired osseointegration and potential effects on jawbone integrity [24].

## Vasoconstrictors:

TCAs, bupropion, maprotiline and herbal agents such as St. John's wort may potentiate the cardiovascular effects of epinephrine, necessitating caution during administration of local anesthesia [25].

## Analgesics:

Acetaminophen may inhibit TCA metabolism, raising the risk of toxicity, while nonsteroidal anti-inflammatory drugs (NSAIDs) can elevate lithium levels and increase bleeding risk when combined with SSRIs [26].

## Antibiotics:

Erythromycin and ciprofloxacin inhibit the metabolism of TCAs and SSRIs, whereas tetracyclines and metronidazole may reduce lithium clearance, predisposing to toxicity [27].

## Antifungals:

Azole antifungals such as ketoconazole and fluconazole, which act as CYP3A4 inhibitors, can increase plasma concentrations of TCAs and trazodone; fluconazole may also potentiate bleeding in patients on valproate [27]. Given these potential interactions, dentists must conduct thorough medication reviews, collaborate with prescribing physicians and carefully adapt anesthetic, analgesic, antibiotic and antifungal regimens to ensure patient safety [24]. These pharmacologic considerations, when combined with behavioral and biological risks, highlight the importance of a comprehensive and individualized dental management approach [25]. Taken together, these biological, behavioral and pharmacologic challenges underscore the need for a comprehensive and preventive approach, which is discussed next [26].

## Strategies for comprehensive dental management of patients on antidepressant therapy:

Management of patients on antidepressants requires balancing medication considerations, mental health assessment and oral health needs [28].

## Medication considerations:

Antidepressants include TCAs, SSRIs, SNRIs, MAOIs and atypical agents [28]. Caution is needed with epinephrine in patients on TCAs or MAOIs due to cardiovascular risks. NSAIDs may increase bleeding and opioids can potentiate sedation. Patients with bipolar disorder often take mood stabilizers (valproic acid, carbamazepine), which may cause hepatotoxicity, thrombocytopenia and immune suppression-necessitating medical consultation before invasive care [29]. Most antidepressant medications are associated with xerostomia, which in turn increases the risk of dental disease. Effective dental management of such patients requires the use of fluoride-based anti-caries agents, saliva substitutes and careful consideration when prescribing or administering analgesics and local anesthetics. Comprehensive dental care not only supports oral health but also contributes significantly to enhancing the patient's self-image and overall quality of life [30]. In geriatric patients, combined cardiovascular and antidepressant therapy can worsen xerostomia and complicate prosthetic treatment [30].

## Mental health assessment:

Before initiating dental treatment, the clinician should establish a strong rapport with the patient and obtain a comprehensive medical history. A non-judgmental approach encourages patients to share relevant details about their condition more openly. By conducting an in-depth medical assessment, the clinician can evaluate the stability of the patient's health status and identify any ongoing symptoms. The presence of such symptoms may indicate inadequate management of the underlying condition or poor adherence to prescribed medications [29]. A confidential and supportive approach helps patients share relevant mental health details. Collaboration with psychiatrists clarifies psychological status and medication regimens. Laboratory tests (liver function, CBC, coagulation) may be warranted before surgical procedures. Dental treatment should be deferred during acute mania or severe depression until stability is restored [31].

## Oral health management:

Xerostomia is common and elevates the risk of caries, periodontal disease and prosthetic complications. Preventive care should include oral hygiene reinforcement, salivary substitutes, restorative management and high-fluoride therapy. Regular 3-month recalls are recommended. Fixed prosthetics are often avoided in elderly patients due to hygiene difficulties, while patient and caregiver education supports long-term outcomes [28]. These approaches not only reduce immediate risks but also highlight the importance of interdisciplinary collaboration and future research to optimize care for this growing patient population [31].

## Conclusion:

Antidepressants, particularly SSRIs and TCAs, are associated with xerostomia, increased caries risk, periodontal irritation and delayed postoperative healing. Depressive disorders may further compromise oral hygiene, compounding these medication-related effects. A coordinated, preventive approach involving medication review, interdisciplinary collaboration and patient education is essential to optimize oral and systemic outcomes in individuals receiving antidepressant therapy.
